# New predictive regression models for estimating the size of unerupted canines and premolars in the Egyptian population

**DOI:** 10.1186/s12903-026-09706-4

**Published:** 2026-09-11

**Authors:** Shaimaa S. EL-Desouky, Shimaa M. Hadwa, Ibrahim A. Kabbash, Safa B. Alawy

**Affiliations:** 1 Pediatric Dentistry, Oral Health, and Preventive Dentistry Department, Faculty of Dentistry, Tanta University, Tanta, Egypt; 2 Public Health & Community Medicine Department, Faculty of Medicine, Tanta University, Tanta, Egypt; 3 Orthodontics Department, Faculty of Dentistry, Tanta University, Tanta, Egypt

**Keywords:** Prediction, Regression equation, Mixed dentition, Premolar, Canine

## Abstract

**Background:**

Mixed dentition space analysis assesses the discrepancy between the available and required space within the dental arches during the mixed dentition stage, thereby facilitating the diagnosis and treatment planning of developing malocclusions. So, this study aimed to develop Egyptian population-specific regression equations for predicting the combined mesiodistal widths of permanent canines and premolars (Σ345) and to compare their predictive accuracy with that of the Tanaka and Johnston method.

**Methods and materials:**

A retrospective cross-sectional study was conducted on 450 dental casts obtained from Egyptian adolescents aged 13–17 years (230 females and 220 males). The mesiodistal crown widths of the maxillary and mandibular permanent incisors, canines, and premolars were measured using a calibrated digital caliper. Multiple linear regression analysis was performed to develop new prediction equations. The predicted widths obtained using the newly developed equations and the Tanaka and Johnston method were compared with the actual tooth measurements.

**Results:**

The mesiodistal widths of the maxillary and mandibular central and lateral incisors demonstrated the strongest correlations with the combined widths of permanent canines and premolars and were therefore used to generate the prediction equations. The newly developed Egyptian equations demonstrated good predictive performance, with coefficients of determination of *R*² = 0.672 for the maxillary arch and *R*² = 0.602 for the mandibular arch. Furthermore, they showed smaller mean prediction errors (0.00 ± 1.03 mm and 0.00 ± 1.34 mm for the maxillary and mandibular arches, respectively) than the Tanaka and Johnston equations (− 1.02 ± 1.22 mm and − 1.30 ± 1.45 mm, respectively).

**Conclusion:**

The newly developed Egyptian-specific regression equations provided more accurate estimates of the mesiodistal widths of unerupted permanent canines and premolars than the Tanaka and Johnston method and may serve as reliable tools for mixed dentition space analysis in Egyptian adolescents.

## Introduction

Predicting the space required for the unerupted canines and premolars is important to the orthodontic evaluation in mixed dentition for deciding treatment alternatives such as timed extractions, eruption guidance, space preservation, regaining space, or even only routine patient monitoring [1, 2]. Accuracy and simplicity are essential standards for a predicting method to be a part of the full case evaluation in contemporary orthodontics [3]. Several methods have been utilized to forecast space for the unerupted teeth [4–7]. The most widely used techniques are Moyers’ probability Table [6] and the prediction equation by Tanaka and Johnston [7].

Moyers’ method utilizes measurements of the four mandibular incisors to estimate the size of unerupted maxillary and mandibular canines and premolars from prediction tables generated for both genders [6]. Numerous studies showed that the total mesiodistal width of the lower permanent incisors isn’t an ideal forecast for determining the mesiodistal widths of unerupted canines and premolars [8–10].

The Tanaka-Johnston method is an easy approach that produces satisfactory results regardless of jaws and sex [11]. It was carried out on North European descent involving 506 North American orthodontic patients; this is questionable for applicability in other populations [12]. Furthermore, the Tanaka–Johnston forecasting equation overestimated the mesiodistal widths of unerupted permanent canines and premolars in the Saudi Arabian [13], Turkish [14], and Bangalore populations [11] since both genetic and environmental variables influence tooth size [15]. Therefore, probability tables and prediction equations for various populations were created [16–18] .

In Egypt, several investigations have been conducted to develop population-specific methods for predicting the mesiodistal widths of unerupted canines and premolars. Aboul Azm and Fouda [19], proposed a prediction approach based on the buccolingual dimensions of the first permanent molars rather than the conventional use of mandibular incisors. Subsequently, Hammad and Abdellatif [1], evaluated mixed dentition space analysis in an Egyptian sample and assessed the applicability of commonly used prediction methods. Later, Ibrahim et al. [20] introduced a digital prediction method for estimating the mesiodistal widths of unerupted canines and premolars, depending on the combination of lower first permanent molars and upper central incisors as the best predictor of canine and premolar widths. Despite these contributions, the reported prediction models differed in the predictor variables used, measurement techniques employed, and study populations investigated. Moreover, the predictive accuracy and clinical applicability of these methods may vary across different Egyptian samples due to variations in tooth-size characteristics and sampling demographics. Therefore, the development and validation of updated regression equations using contemporary Egyptian data remain important to improve the accuracy and reliability of mixed dentition space analysis.

Accordingly, the present study aimed to develop new Egyptian-specific regression equations for predicting the combined mesiodistal widths of permanent canines and premolars in both arches using a large contemporary sample of Egyptian adolescents. Unlike previous Egyptian studies that used different predictor variables or smaller and older datasets, the present study developed updated regression equations using a large contemporary sample of Egyptian adolescents.

The predictive performance of the newly developed equations was subsequently evaluated and compared with that of the widely used Tanaka and Johnston method. The null hypothesis (H₀) stated that no significant differences would exist between the actual mesiodistal widths of the permanent canines and premolars and the widths predicted by either the newly developed Egyptian regression equations or the Tanaka and Johnston method in the Egyptian population.

## Materials & methods

### Study setting and ethical considerations

A retrospective, cross-sectional study was conducted at the Outpatient Clinics of Paediatric Dentistry and Orthodontic Departments, Faculty of Dentistry, Tanta University. This study was approved by the ethical committee (REC), Faculty of Dentistry, Tanta University, code (#R-PED-5-24-3107), complying with the Helsinki Declaration of 1964 and its subsequent amendments. As all participants were adolescents aged 13–17 years, written informed consent to participate was obtained from the parents or legal guardians of all participants at the time of their initial attendance at the outpatient clinics, together with assent from the adolescent participants. Consent included permission to use anonymized dental casts and clinical records for future retrospective research purposes.

### Eligibility criteria

A total of 1,045 pairs of maxillary and mandibular pre-treatment dental casts belonging to Egyptian adolescents aged 13–17 years were retrieved from the archives of the Outpatient Clinics of the Paediatric Dentistry and Orthodontic Departments, Faculty of Dentistry, Tanta University. The casts were screened according to predefined inclusion and exclusion criteria. The inclusion criteria comprised dental casts with fully erupted permanent teeth from the right first permanent molar to the left first permanent molar in each arch, free from developmental morphological anomalies and conditions that could affect the accuracy of tooth measurements.

The exclusion criteria included casts from adolescents presenting with crowding, malaligned teeth, abnormalities in tooth number (congenitally missing teeth, retained primary teeth, or supernumerary teeth), proximal caries, extensive restorations, enamel hypoplasia, fixed or temporary crowns, attrition, abrasion, previous orthognathic surgery, ongoing or previous orthodontic treatment, cleft palate, palatal asymmetries, systemic disorders, or craniofacial syndromes known to affect dental development (e.g., ectodermal dysplasia). Casts with technical defects, such as fractures or air bubbles affecting tooth morphology, were also excluded.

**Fig. 1:**
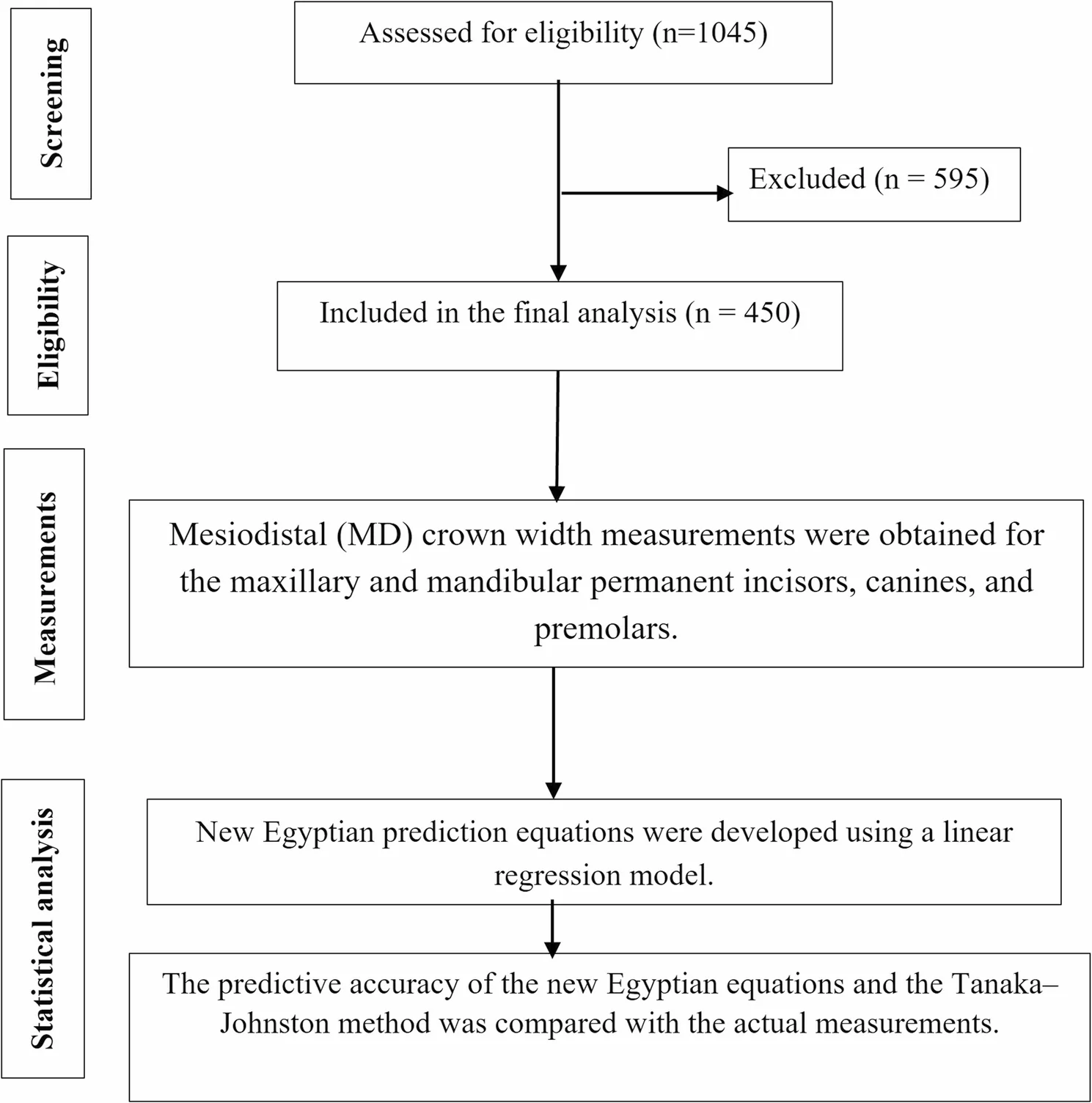
Flowchart illustrating cast screening, eligibility assessment, dental measurements, and statistical analysis

Following eligibility assessment, 595 casts were excluded, and 450 casts fulfilled the selection criteria and were included in the final analysis. Figure 1 illustrates the process of cast screening, eligibility assessment, dental measurements, and statistical analysis.

### Sample size calculation

The sample size was calculated using Epi Info™ version 7.2.7 (World Health Organization and Centers for Disease Control and Prevention, Atlanta, Georgia, USA). Assuming an expected proportion of 75%, a 95% confidence level, and a margin of error of 7%, the minimum required sample size was estimated to be 447 participants. To compensate for incomplete records and enhance data quality, the sample size was increased to 450 participants.

### Mesiodistal crown measurements

The mesiodistal (MD) crown widths of the maxillary and mandibular permanent incisors, canines, and premolars were measured on dental casts using a calibrated digital caliper (0–200 mm, accuracy 0.1 mm; Carbon Fiber Digital Vernier Caliper, Future Electronics, Egypt, https://store.fut-electronics.com). The greatest mesiodistal crown width of each tooth was measured between the anatomical contact points with the caliper positioned parallel to the occlusal plane and perpendicular to the long axis of the tooth, as described by Moorrees et al. [5]. Measurements were obtained for homologous teeth on both sides of each arch, and the mean value of the right and left measurements was used for subsequent analyses.

To minimize measurement error, all measurements were performed in millimetres by a single experienced and calibrated examiner (last author), who examined a maximum of 10 pairs of casts per day to reduce eye fatigue [21]. Each measurement was repeated three times, and the mean value was recorded. Demographic data, including age and sex, were obtained from the corresponding patient records.

Intra-examiner reliability was assessed by repeating measurements on 10% of the sample after a one-month interval. The intraclass correlation coefficient (ICC) was 0.86, indicating good intra-examiner reliability.

### Statistical analysis

Data were collected, tabulated, and analyzed using IBM SPSS Statistics for Windows, Version 19.0 (IBM Corp., Armonk, NY, USA). Descriptive statistics were calculated and presented as mean ± standard deviation (SD), median, interquartile range (IQR), minimum, and maximum values, as appropriate. Differences in the mean mesiodistal widths of the measured teeth between males and females were assessed using the independent-samples Student’s t-test.

Pearson’s correlation coefficient (r) was used to evaluate the association between the mesiodistal crown widths of the maxillary and mandibular incisors and the combined mesiodistal widths of the permanent canines and premolars. Variables showing the strongest correlations were subsequently entered into multiple linear regression analyses to develop prediction equations for estimating the combined mesiodistal widths of the permanent canines and premolars in the maxillary and mandibular arches.

The predictive performance of the newly developed Egyptian equations was assessed by comparing the predicted values with the actual measured widths and with the values predicted by the Tanaka and Johnston method. Pearson’s correlation coefficient was also used to evaluate the relationship between the actual widths and those predicted by both methods. Statistical significance was set at *p* < 0.05.

## Results

A total of 450 dental casts were included in the study, comprising 230 (51.1%) females and 220 (48.9%) males. The mean age of the female and male participants was 14.93 ± 0.67 years and 14.76 ± 0.45 years, respectively. No statistically significant sex-related differences were observed in the mesiodistal widths of the measured teeth (Table 1).

**Table 1:** Comparison of mesiodistal width of the measured teeth of the upper and lower arch by gender

Variables	Males	Females	t	*P*
Upper arch				
Upper central	8.02 ± 0.86	8.00 ± 0.89	0.241	0.809
Upper lateral	6.16 ± 0.73	6.10 ± 0.86	0.809	0.419
Upper canine	7.16 ± 0.77	7.10 ± 0.74	0.841	0.401
Upper 4	6.30 ± 0.53	6.25 ± 0.78	0.723	0.470
Upper 5	6.07 ± 0.50	6.00 ± 0.73	1.185	0.237
Lower arch				
Lower central	4.47 ± 0.55	4.59 ± 0.69	–1.946	0.052
Lower lateral	4.90 ± 0.53	4.95 ± 0.63	–0.903	0.367
Lower canine	6.19 ± 0.81	6.13 ± 0.73	0.903	0.367
Lower 4	6.26 ± 0.74	6.19 ± 0.95	0.868	0.386
Lower 5	6.26 ± 0.74	6.19 ± 0.95	0.874	0.383

### Establishment of new prediction Egyptian-specific equations

In the maxillary arch, the mesiodistal widths of canines and premolars (∑345) had the strongest association with the mesiodistal widths of maxillary central incisors (*r* = 0.737) and maxillary lateral incisors (*r* = 0.778). Also, in the mandible, the strongest correlation was observed between the total mesiodistal width of canines and premolars and the mesiodistal widths of the mandibular central incisors (*r* = 0.682) and mandibular lateral incisors (*r* = 0.751) (Table 2).

**Table 2:** Correlation of mesiodistal crown width of measured teeth of the upper and lower arch

Variables	Maxillary arch			
	Mesiodistal upper central incisor		Mesiodistal upper lateral incisor	
	*R*	*P*	*r*	*p*
Upper canine	0.644	< 0.001*	0.708	< 0.001*
Upper 4	0.627	< 0.001*	0.653	< 0.001*
Upper 5	0.667	< 0.001*	0.683	< 0.001*
Sum of upper canine, 4 & 5	0.737	< 0.001*	0.778	< 0.001*

	**Mandibular arch**			
	**Mesiodistal lower central**		**Mesiodistal lower lateral**	
	***R***	***P***	***r***	***p***
Lower canine	0.569	< 0.001*	0.632	< 0.001*
Lower 4	0.672	< 0.001*	0.714	< 0.001*
Lower 5	0.673	< 0.001*	0.714	< 0.001*
Sum of lower canine, 4 & 5	0.682	< 0.001*	0.751	< 0.001*

*Significant

Based on the regression analysis, the mesiodistal widths of the maxillary and mandibular central and lateral incisors were identified as the strongest predictors of the combined mesiodistal widths of the permanent canines and premolars. Accordingly, two arch-specific prediction equations were developed (Table 3):

**Table 3:** Linear regression characteristics of the new prediction Egyptian equations for the maxillary and mandibular arches

Variables	Maxillary arch				
	B	Standardized B	SE	t	*p*
Constant	6.194	-------	0.455	13.604	< 0.001*
Mesiodistal upper central	0.763	0.370	0.079	9.644	< 0.001*
Mesiodistal upper lateral	1.163	0.515	0.087	13.411	< 0.001*
Prediction equation	Σ345 = 6.194 + 0.763(UCI) + 1.163(ULI)				

Variables	Mandibular arch				
	B	Standardized B	SE	t	*p*
Constant	4.506	------	0.546	8.245	< 0.001*
Mesiodistal lower central	0.992	0.291	0.147	6.754	< 0.001*
Mesiodistal lower lateral	1.961	0.540	0.157	12.509	< 0.001*
Prediction equation	Σ345 = 4.506 + 0.992(LCI) + 1.961(LLI)				

*Significant, *UCI *Upper Central Incisor width, *ULI *Upper Lateral Incisor width *R*^2^ = 0.672, F= 461.84, *p* <0.001Estimated error of the estimate = 1.025

*Significant, LCI (Lower Central Incisor width), LLI (Lower Lateral Incisor width) *R*^2^ = 0.602, F= 340.85, *p* <0.001Estimated error of the estimate = 1.337

#### Maxillary arch

$$\textstyle\sum{345=6.194 + 0.763}\left(\mathrm{UCI}\right)+1.163\left(\mathrm{ULI}\right)$$

#### Mandibular arch

$${\textstyle\sum}345=4.506+0.992\left(\mathrm{LCI}\right)+1.961\left(\mathrm{LLI}\right)$$

where Σ345 represents the combined mesiodistal width (mm) of the permanent canine, first premolar, and second premolar within one quadrant, UCI represents upper central incisor width, ULI represents upper lateral incisor width, LCI represents lower central incisor width, and LLI represents lower lateral incisor width. All predictor measurements are expressed in millimetres.

### Accuracy of the new Egyptian equations compared to Tanaka and Johnston equations

In both arches, significant positive correlations were observed between the actual combined mesiodistal widths of the permanent canines and premolars and the widths predicted by both the newly developed Egyptian equations and the Tanaka and Johnston equations (*p* < 0.001) (Table 4).

**Table 4:** The correlation coefficients in both arches between the actual sum of the canine and premolar mesiodistal widths and those forecasted by the Tanaka and Johnston equations and the new Egyptian equations

Variables	Actual MD sum of canine, 4 & 5			
	Maxillary arch		Mandibular arch	
	*R*	*p*	*R*	*p*
Egyptian equation	0.821	< 0.001	0.777	< 0.001
Tanaka & Johnston	0.744	< 0.001	0.770	< 0.001

Table 5 presents the differences between the actual and predicted mesiodistal widths of the permanent canines and premolars in the study sample. The newly developed Egyptian equations demonstrated smaller mean prediction errors (maxillary: 0.00 ± 1.03 mm; mandibular: 0.00 ± 1.34 mm) than the Tanaka and Johnston equations (maxillary: − 1.02 ± 1.22 mm; mandibular: − 1.30 ± 1.45 mm), indicating closer agreement with the actual tooth measurements (Table 5).

**Table 5:** Comparison of differences between the actual sum of the mesiodistal widths of canines and premolars and those estimated by the new Egyptian equations and Tanaka and Johnston equations in both arches

Variables		New Egyptian equation	Tanaka & Johnston equation
Maxillary arch	Range	–2.73 to 3.24	–4.40 to 3.50
	Mean ± SD	0.00 ± 1.03	–1.02 ± 1.22
	Median	0.00	–1.00
	IQ range	–0.65 to 0.61	–1.90 to − 0.10
Mandibular arch	Range	–4.55 to 4.71	–5.50 to 3.00
	Mean ± SD	0.00 ± 1.34	–1.30 ± 1.45
	Median	0.02	–1.40
	IQ range	–0.89 to 0.79	–2.20 to − 0.40

**Fig. 2:**
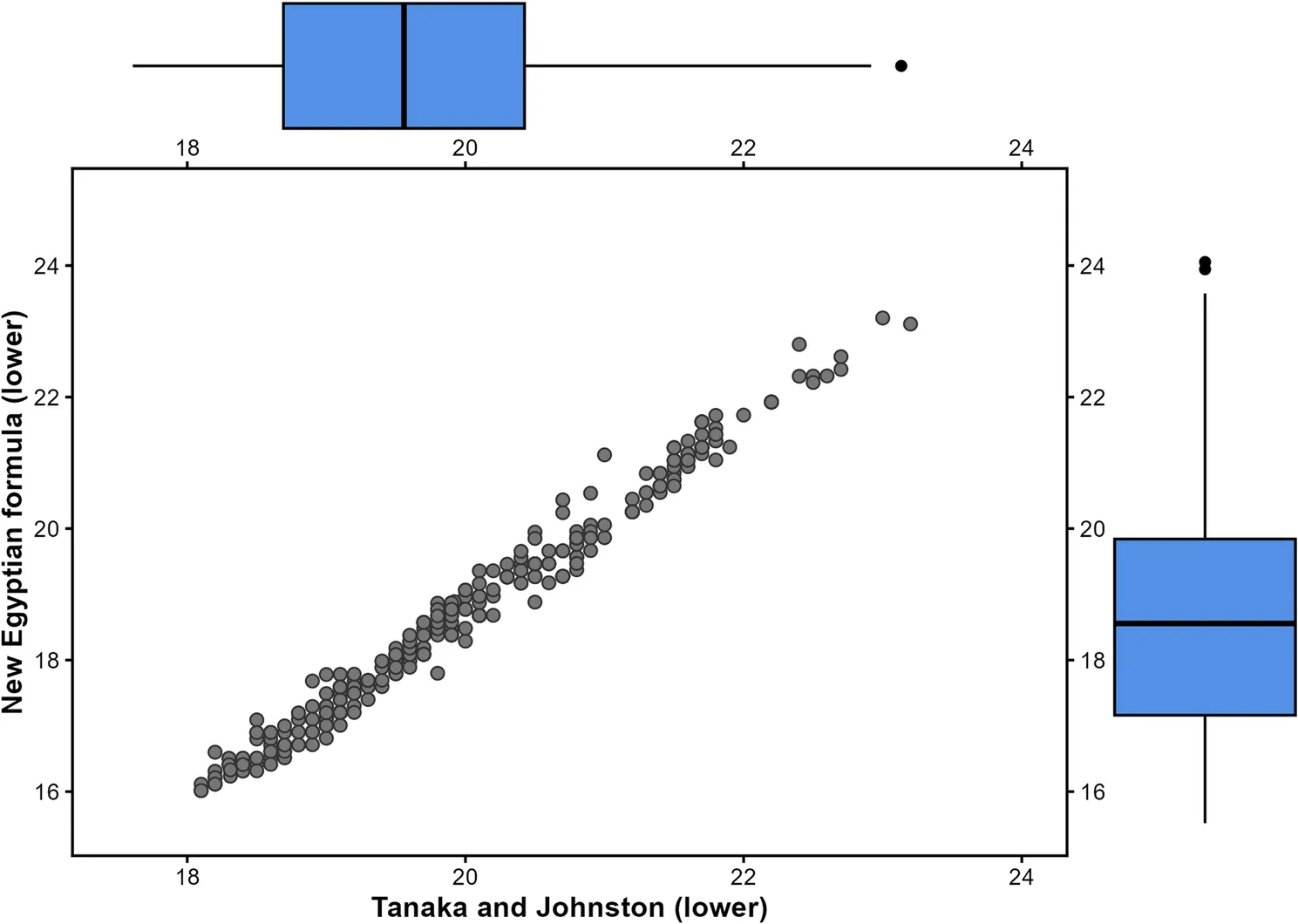
Regression plot of Tanaka & Johnston and the Egyptian formula of mandibular arch

**Fig. 3:**
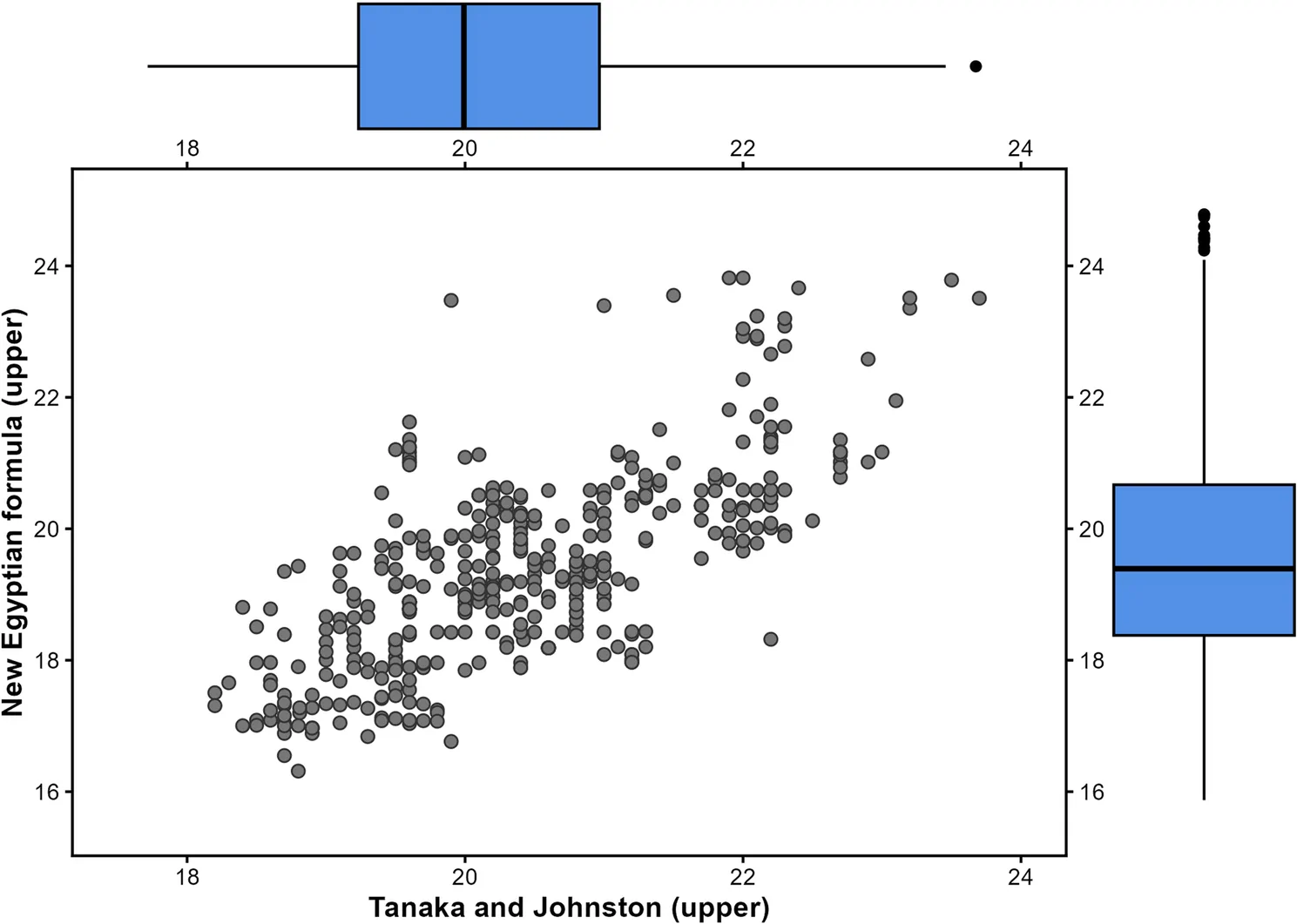
Regression plot of Tanaka & Johnston and the Egyptian formula of maxillary arch

In the maxillary arch, the mean prediction error obtained using the new Egyptian equation was lower than that observed in the mandibular arch. Furthermore, the newly developed Egyptian equations showed negligible prediction bias, whereas the Tanaka and Johnston equations consistently underestimated the actual mesiodistal widths of the permanent canines and premolars in both arches (Table 5; Figs. 2 and 3). 

## Discussion

The mesiodistal widths of permanent teeth vary by race and ethnicity [22], therefore, data gathered from a single ethnicity to forecast the size of unerupted permanent teeth may not be appropriate for another race [23]. Therefore, the present study aimed to create simpler, more reliable equations that might be applied to forecast the unerupted canine and premolar widths in Egyptian children. This study adopted a non-radiographic approach (simple regression equation) for mixed dentition analysis, as the radiographic methods were comparatively inaccurate, in addition to the patient’s awareness of radiation exposure hazards [12]; this is in accordance with Flores-Mir et al. [24] who found that using traditional panoramic scans, the tooth length measurements were overestimated by 29%; additionally, the lengths were 4% underestimated by CBCT panoramic reconstructions.

In the current study, the mesiodistal crown widths of mandibular and maxillary incisors were selected to forecast the widths of the mandibular and maxillary canines and premolars due to their early eruption in the oral cavity; this agreed with Eliewy Saloom et al. [18] who used the sum mesiodistal widths of the mandibular and maxillary incisors and first molars to predict, with high accuracy (86%), the combined mesiodistal widths of the mandibular and maxillary canines and premolars. A dental plaster cast with a digital caliper was utilized in this study for mesiodistal crown measurements, as it is an efficient, precise method; this concurred with Correia et al. [25], who found no significant differences between the values calculated by digital and manual methods.

The present study found no statistically significant sex differences in tooth widths. This finding contrasts with those of Chong et al. [12] and Eliewy Saloom et al. [18], who reported significantly larger tooth dimensions in males than females. Similarly, Ling and Wong [26] found that males exhibited greater mesiodistal tooth widths than females for all tooth types, except the mandibular lateral incisors.

The absence of sexual dimorphism in the present study may reflect population-specific characteristics, as tooth dimensions and the degree of sexual dimorphism vary among ethnic groups [27]. The relatively narrow age range (13–17 years) and homogeneous sample may also have contributed to the lack of significant sex-related differences. This finding is consistent with Paulino et al. [28], who reported no significant sex differences in mesiodistal tooth dimensions among Spanish adolescents, although differences were observed in older age groups. Overall, variations in genetic background, age, sample characteristics, and measurement methods may account for the inconsistent findings regarding sexual dimorphism across populations.

The null hypothesis was rejected, as the newly developed Egyptian equations demonstrated closer agreement with the actual tooth dimensions than the Tanaka and Johnston method. The strong associations between incisor widths and the combined widths of canines and premolars support the use of erupted incisors as predictors in mixed-dentition analysis. Similar relationships have been reported in Jordanian [29], Turkish [14], Ugandan [17], and Taiwanese [12] populations, although differences in correlation strength may reflect ethnic variations in tooth morphology and dimensions.

The newly developed equations showed smaller mean prediction errors than the Tanaka and Johnston method in both arches, indicating closer agreement with the actual measurements. The mean prediction errors were close to zero for the Egyptian equations, suggesting negligible prediction bias, whereas the negative mean errors obtained with the Tanaka and Johnston method indicate a tendency toward underestimation in the present Egyptian sample. This finding is consistent with Chong et al. [12], who reported that the Tanaka–Johnston method underestimated the widths of unerupted canines and premolars in a Taiwanese population. Similarly, Melgaco et al. [30] reported underestimation of the lower canine and premolar widths in Brazilian males. Conversely, Arslan et al. [14] and Buwembo et al. [17] reported overestimation using the Tanaka–Johnston method in Turkish and Ugandan populations, respectively. These differences may be related to ethnic variation, sample characteristics, and methodological differences.

The predictive models explained 67.2% of the variance in the combined mesiodistal widths of the maxillary canines and premolars and 60.2% of the variance in the mandibular arch. Prediction performance was slightly better in the maxillary than in the mandibular arch, which contrasts with the findings of Paredes et al. [31], who reported greater predictive accuracy in the mandibular arch in a Spanish population. Differences in population characteristics, study design, predictor variables, and evaluation criteria may explain this discrepancy.

Although the newly developed Egyptian equations showed negligible prediction bias, the clinical implications of prediction error should be considered. In general, slight overestimation may be preferable to underestimation because it reduces the risk of inadequate space and subsequent crowding; however, excessive overestimation may lead to unnecessary tooth extractions [18, 32]. Nahidh [33] and Eliewy Saloom et al. [18] reported that an overestimation of up to 1 mm per side of the dental arch is unlikely to influence extraction decisions. Therefore, the small prediction errors observed with the newly developed equations are unlikely to have a substantial impact on clinical decision-making.

The present findings extend the evidence on mixed dentition prediction in Egyptian populations by providing updated regression equations derived from a large contemporary sample of Egyptian adolescents. The equations, based on the strongest predictors identified through regression analysis, demonstrated high predictive performance in both arches. Compared with the widely used Tanaka and Johnston method, they showed smaller mean prediction errors and closer agreement with actual tooth dimensions. The high coefficients of determination (*R*² = 0.672 for the maxillary arch and *R*² = 0.602 for the mandibular arch) further support the reliability and potential clinical applicability of these population-specific equations for mixed-dentition analysis.

Although the proposed equations demonstrated high predictive accuracy in the present sample, external validation in independent Egyptian populations from different geographic regions is needed before widespread clinical application. Future studies should compare their performance with existing Egyptian prediction models and other commonly used methods to further assess their generalizability and clinical applicability across diverse Egyptian populations.

The major limitation of the present study was its cross-sectional design; therefore, a longitudinal study is recommended to monitor sequential developmental changes, obtain accurate dimensions, and subsequently compare predicted measurements with actual values. Also, this study compared the predicted measurements with only the Tanaka and Johnston equations; further studies can be directed to compare with other commonly used methods. In addition, studies involving growing children with different skeletal growth patterns are warranted to further validate the proposed prediction models and enhance their clinical applicability across diverse craniofacial growth patterns.

## Conclusion

Based on the findings of this study, the newly developed Egyptian-specific regression equations demonstrated closer agreement with the actual combined mesiodistal widths of permanent canines and premolars than the Tanaka and Johnston method in the studied Egyptian sample. The equations showed negligible mean prediction bias and smaller mean prediction errors in both arches, with slightly better predictive performance in the maxillary arch (*R*² = 0.672) than in the mandibular arch (*R*² = 0.602). These population-specific equations provide a simple approach for estimating the widths of unerupted canines and premolars and may be useful for mixed-dentition space analysis in Egyptian adolescents. However, external validation in independent Egyptian populations is recommended before widespread clinical application. 

## Data Availability

The datasets used and/or analyzed during this study are available from the corresponding author upon reasonable request.
